# C-954-08. Impact of Temperature on Burn Operating Room Demand at a Regional Burn Center

**DOI:** 10.1093/jbcr/irag033.140

**Published:** 2026-04-06

**Authors:** Brenna Chen, Paul P Kim, Vidhani Goel, Alexander Rowan, Kevin Gladin, Lateef Omidiji Jr., Shane Jung, Kavita Batra, Rabia Nizamani, Stephanie Martinez

**Affiliations:** Leon S. Peters Burn Center, Las Vegas, NV; Leon S. Peters Burn Center, Las Vegas, NV; Kirk Kerkorian School of Medicine at University of Nevada, Las Vegas, NV; Leon S. Peters Burn Center, Las Vegas, NV; University Medical Center Lions Burn Care Center, Las Vegas, NV; Kirk Kerkorian School of Medicine at University of Nevada, Las Vegas, NV; Leon S. Peters Burn Center, Las Vegas, NV; Kirk Kerkorian School of Medicine at University of Nevada, Las Vegas, NV; University Medical Center Lions Burn Care Center, Las Vegas, NV; University of Nevada Las Vegas, Las Vegas, NV

## Abstract

**Introduction:**

High ambient outdoor temperatures influence heat related injuries, but less is known about seasonal effects on burn-related operating room (OR) demand. This study aims to investigate effects of daily temperature on burn OR cases and OR time, accounting for seasonality, variability, and lagged impacts.

**Methods:**

Burn OR case counts and OR time data was collected from the burn registry at a single regional burn center and matched with daily temperature data from local meteorological sources. Hinge (piecewise) linear regression models were used to identify a temperature threshold where OR demand rises. To evaluate the temporal dynamics, we constructed lagged hinge terms (0–14 days) to identify the delay between exceeding the threshold and observed increases in OR demand.

**Results:**

Descriptively, the average daily number of burn OR cases was 2.22 (SD = 1.53), with annual totals ranging from 709 to 939 cases. Average daily burn OR cases was 2.22 (SD = 1.53). Seasonal averages were highest in summer (2.86 cases/day) and lowest in spring (1.81 cases/day) (Fig. 1). Average daily OR time followed a similar pattern (summer = 6.5 hrs, spring = 3.79 hrs) (Fig. 2). Holidays were associated with 0.74 fewer cases per day (*p*<.001) and 1.22 fewer OR hours per day (*p*=.004). However, summer holidays (e.g., July 4th) and winter holidays (e.g., New Years) were associated with visible surges in OR demand. Hinge regression analysis identified a temperature threshold of 57°F, above which case counts rose with each degree of increase (+0.02 cases/°F). OR time saw a similar pattern, rising by 0.30 hours per °F above threshold (*p*=.01) (Fig. 3). Finally, lagged models indicated the best fit at 8–11 days, suggesting that increases in burn OR demand are most evident 1-2 weeks after threshold exceedance.

**Conclusions:**

Burn OR cases and time at our center show a clear temperature threshold at ~57°F, with activity rising above this point (0.02 cases and 0.3 operative hours per °F) and peaking after an ~11-day lag. Though daily variability appears less important, there is a clear seasonal demand for burn surgery needs that correlate with the ebb and flow of temperature according to seasons, showing a predictive utility in monitoring temperatures to allocate resources accordingly.

**Applicability of Research to Practice:**

While this data is specific to our institution’s desert climate, these results highlight lagged climate-health connections that may aid in operational planning and resource allocation at other burn centers.

**Funding for the Study:**

N/A.

